# *Fgf21* Deficiency Delays Hair Follicle Cycling and Modulates miRNA–Target Gene Interactions in Mice

**DOI:** 10.3390/biology14050526

**Published:** 2025-05-09

**Authors:** Yana Li, Yue Ao, Xinru Xie, Tug Ulan, Dongjun Liu, Xudong Guo

**Affiliations:** The State Key Laboratory of Reproductive Regulation and Breeding of Grassland Livestock, Inner Mongolia University, Hohhot 010031, China; 15391171312@163.com (Y.L.); 15326025339@139.com (Y.A.); 15147635442@163.com (X.X.); wlantuge@163.com (T.U.); nmliudongjun@sina.com (D.L.)

**Keywords:** *Fgf21*, hair follicle, microRNAs, target genes

## Abstract

Fibroblast growth factor 21 (*Fgf21*) is a key regulator of hair follicle development and cycling. Although microRNAs (miRNAs) are involved in this process, the specific mechanisms through which *Fgf21* regulates hair follicle growth remain unclear. This study investigates the role of *Fgf21* and its associated miRNAs in hair follicle growth and development. Using CRISPR/Cas9, we generated *Fgf21* knockout mice (*Fgf21^−^/^−^*), which exhibited a delayed transition from the telogen to anagen phases compared to wild-type (WT) mice. miRNA sequencing identified differentially expressed miRNAs in *Fgf21*^−^/^−^ mice, with dual-luciferase assays confirming that miR-134-5p directly targets vascular endothelial zinc finger 1 (Vezf1) and miR-136-5p targets mitogen-activated protein kinase kinase kinase 1 (Map3k1). Real-time qPCR analysis revealed that Vezf1 and Map3k1 expression was higher in *Fgf21^−^/^−^* mice than in WT mice during catagen, but lower during telogen. These findings indicate that *Fgf21* plays a critical role in regulating hair follicle growth and may modulate Vezf1 and Map3k1 expression through miRNAs. This study provides novel insights into the molecular regulation of hair follicle growth and suggests potential therapeutic strategies for hair follicle-related disorders.

## Simple Summary

This study explores how *Fgf21* gene influences hair follicle growth. Using CRISPR/Cas9, we developed *Fgf21* gene knockout mice, which showed a delayed shift from the telogen to anagen phase of the hair follicle cycle compared to normal mice. Through miRNA sequencing, we found specific miRNAs that are differentially expressed in these knockout mice. Further experiments confirmed that miR-134-5p directly targets the *Vezf1* gene, and miR-136-5p targets the *Map3k1* gene. These genes are involved in regulating hair follicle development. Our findings suggest that *Fgf21* gene plays a crucial role in hair follicle growth by modulating the expression of target genes through miRNAs. This research deepens our understanding of the molecular mechanisms underlying hair follicle growth and could aid in developing novel therapies for hair-related disorders.

## 1. Introduction

Hair follicles are essential skin appendages that arise from the interactions between cells of different origins, including epithelial, mesenchymal, and neurodermal cells [1]. Hair follicles serve as the foundation for hair growth and provide structural support and protection to the skin [2]. Hair follicle formation is a complex process involving the coordinated actions of surrounding tissues and cells and is regulated by multiple signaling pathways and transcription factors [3].

In recent years, the molecular mechanisms underlying hair follicle development have gained considerable attention. The fibroblast growth factor (FGF) family plays a crucial regulatory role in this process. The FGF family comprises a large group of growth factors that regulate cell proliferation, apoptosis, migration, and differentiation [4,5]. Dysregulation of FGF family genes significantly affects hair follicle growth and development. For instance, *Fgf18* is considered a key regulator of the hair follicle growth cycle, as *Fgf18* knockout mice exhibit shortened telogen [6], whereas *Fgf7* overactivation markedly inhibits hair follicle growth [7]. *Fgf20* is essential for early dermal condensate formation [8,9], whereas *Fgf9* and *Fgfr2 IIIb* are involved in hair follicle regulation [10,11]. Interactions between Fgf and Wnt signaling in the mesenchymal niche regulate the hair follicle cycle clock in mice [12], and *Fgfr2* negatively regulates sonic hedgehog (*Shh*) during hair follicle development [13]. Members of the FGF and FGFR families regulate the entire hair follicle growth cycle in mice, with different genes expressed at various stages. During anagen, *Fgf21* and its receptor *Fgfr2* are expressed; during catagen, *Fgfr1* and *Fgfr4* are expressed; and during telogen, *Fgf1*, *Fgf2*, *Fgf13*, *Fgf18*, and *Fgf20* are expressed [14]. These findings highlight the critical role of the FGF family in hair follicle growth and development.

*Fgf21* was first isolated in 2000 by Nishimura et al. [15] and belongs to the FGF19 subfamily, which also includes *Fgf19*, *Fgf21*, and *Fgf23* [16]. While *Fgf21* has been recognized as a key regulator of hair follicle growth and development, its role in the miRNA regulatory network, particularly in the context of hair follicle growth and cell cycle regulation, remains underexplored. Therefore, elucidating the specific mechanisms by which *Fgf21* regulates hair follicle growth and development, as well as its role in hair follicle cell cycle regulation, is crucial for understanding hair follicle biology and may provide new therapeutic strategies for related disorders.

MicroRNA sequencing technology has revealed the significant role of miRNAs in hair follicle growth, development, and transition between hair follicle cycles [17]. To investigate the role of *Fgf21* in hair follicle growth and cycle regulation, our laboratory used CRISPR/Cas9 to develop a *Fgf21* knockout mouse model. High-throughput miRNA sequencing found 93 differentially expressed miRNAs, with 10 selected for their potential roles in hair follicle regulation; *Vezf1* and *Map3k1* were confirmed as direct targets of miR-134-5p and miR-136-5p, respectively, and their expression within follicles was altered by *Fgf21* during the first natural growth cycle of mouse hair follicles. These findings indicate that *Fgf21* knockout induces changes in the miRNA expression profile and regulates *Vezf1* and *Map3k1* expression, thereby influencing hair follicle growth and development.

## 2. Materials and Methods

### 2.1. Animal Models and Sample Collection

This study used FVB strain, an outbred strain derived from Swiss mice and developed by the National Institutes of Health from the Friend Virus Susceptibility 1b (FV1b) inbred strain. We established a *Fgf21* knockout mouse model using CRISPR/Cas9 technology. The mouse model was bred through intercrosses and reciprocal crosses of heterozygous and homozygous mice. All experimental animals were housed in a controlled environment with a temperature of 22–24 °C, humidity of 45–65%, and free access to food and water. Genomic DNA was extracted from mouse tail tips using a genomic DNA extraction kit (Tiangen Bio, Beijing, China) to confirm successful *Fgf21^−^/^−^* model establishment. Genotyping was performed via PCR using the following primers: *Fgf21*-F:5′-GTTCCTGCCAAGTGTGTCAAATA-3′; *Fgf21*-R:5′-GACCACAGAAAGAGACTCACCG-3′. The reaction procedure was as follows: initial denaturation at 94 °C for 5 min, followed by 35 cycles of denaturation at 94 °C for 30 s, annealing at 56 °C for 30 s, and extension at 72 °C for 50 s. Finally, the reaction was held at 72 °C for 10 min and then cooled to 16 °C for 1 h. The genotype identification results are listed in Figure S1 in the Supplementary Materials.

Dorsal skin tissues of 8-week-old FVB *Fgf21^−^/^−^* and WT mice were collected for miRNA sequencing. All animal experiments were conducted in strict accordance with the Guidelines for the Management and Use of Laboratory Animals established by the Laboratory Animal Research Center of Inner Mongolia University (SYXK2020-0006). The animal experiments in this study were approved and authorized by the Animal Ethics Committee of Inner Mongolia University (IMU-MOUSE-2020-047). These measures ensured scientific rigor and ethical compliance, providing a reliable model for investigating the role of *Fgf21* in the hair follicle growth cycle.

### 2.2. Preparation of Hair Follicle Cycle-Synchronized Mouse Model

To assess the role of *Fgf21* in the hair follicle growth cycle, we synchronized the hair follicle cycle in 8-week-old FVB mice using a physical depilation method. After inducing anesthesia, a mixture of rosin and paraffin was applied to the dorsal skin to induce depilation. Subsequently, we compared the hair growth rates between WT and *Fgf21^−^/^−^* mice. Hair growth was monitored and photographed at 1-, 3-, 5-, 7-, 9-, 11-, 13-, 15-, 17-, 19-, and 21-days post-depilation to document the process in detail.

8-week-old mice are in the telogen phase of the hair follicle cycle [18]. After depilation, hair follicles synchronously transition to the anagen phase [19]. We use the model to study the transition from telogen to anagen in mice. For target gene validation in the hair follicle cycle, naturally cycling mice are selected. On postnatal day 6 (P6), hair follicles are in the anagen phase, characterized by active cell proliferation and hair shaft formation, while P5 is regarded as early anagen due to the continuity of follicular development [20]. P15 and P17 mice are in the catagen phase, which lasts until P19, followed by the resting telogen phase, with P20 being in early telogen [20]. Based on this information, skin tissue from P5 mice represents anagen, P16 represents catagen, and P20 represents telogen.

### 2.3. Real-Time Quantitative PCR (RT-qPCR)

In the RT-qPCR experiment, total RNA was extracted from fresh frozen samples. After collection, the samples were stored in liquid nitrogen tanks and RNA extraction was performed once all samples were collected. Total RNA was extracted from the dorsal skin tissues of 8-week-old WT and *Fgf21^−^/^−^* mice using the RNAiso Plus reagent kit (Takara Bio Inc., Tokyo, Japan). The extracted RNA was reverse transcribed into cDNA using the PrimeScript Reverse Transcriptase Kit (Takara Bio Inc., Tokyo, Japan). Reverse transcription was performed with the GoScript Reverse Transcription System (Promega Corporation, Madison, WI, USA) on a C1000 Thermal Cycler (Bio-Rad Laboratories Inc., Hercules, CA, USA). RT-qPCR reactions were conducted on the C1000 Thermal Cycler using the GoTaq qPCR Master Mix (Promega Corporation, Madison, WI, USA). GAPDH and U6 were selected as internal reference genes to ensure the accuracy and comparability of the data. The primer sequences used for RT-qPCR are listed in Table S1 in the Supplementary Materials. Relative gene expression levels were calculated using the 2^−ΔCT^ method, based on the difference between the CT values of the target genes and the reference genes.

### 2.4. miRNA Library Construction and High-Throughput Sequencing

miRNA sequencing libraries were constructed from the total RNA extracted from the fresh dorsal skin tissues of 8-week-old FVB mice using the TruSeq Small RNA Sample Preparation Kit (Illumina Inc., San Diego, CA, USA). PCR products of 140–160 bp were recovered using 6% polyacrylamide gel electrophoresis for library preparation. Single-end high-throughput sequencing was performed on an Illumina HiSeq 2000/2500 platform (Illumina Inc., San Diego, CA, USA).

### 2.5. Data Quality Control and miRNA Identification

The quality of raw miRNA sequencing data was assessed using Illumina FastQC (Illumina Inc., San Diego, CA, USA), and high-quality sequences meeting the Q30 standard were selected for further studies. These sequences were aligned to the miRBase 21.0 database (Memorial Sloan-Kettering Cancer Center, New York, NY, USA) using Bowtie (Johns Hopkins University, Baltimore, MD, USA) to identify and quantify known miRNAs. Sequences that did not match the known miRNAs were further aligned with the reference genome. The secondary structure of the successfully aligned sequences was predicted using RNAfold (University of Vienna, Vienna, Austria) to identify and analyze the structural features of the miRNAs.

### 2.6. Differential Expression Analysis of miRNAs

The expression levels of miRNAs were analyzed using RSEM (Harvard Medical School, Boston, MA, USA) based on sequencing data, with expression values normalized to transcripts per million (TPM). Differential expression analysis of the miRNAs was performed using DESeq2 (Bioconductor, Heidelberg, Germany). DESeq2 employs a negative binomial distribution model to evaluate the statistical significance of miRNA expression differences while accounting for sample variability. The analysis was performed using default parameters, a significance level of *p*-value < 0.05, and a fold-change cutoff of 2 for identifying upregulated and downregulated miRNAs. The Benjamani–Hochberg method was applied for multiple testing correction to enhance accuracy.

### 2.7. Target Gene Enrichment Analysis and Network Construction

To identify the target genes of differentially expressed miRNAs associated with hair follicle growth and development, we employed three prediction tools: TargetScan 8.0 (Whitehead Institute for Biomedical Research, Cambridge, MA, USA), miRanda 2.066 (Memorial Sloan-Kettering Cancer Center, New York, NY, USA), and miRDB 6.0 (University of Illinois, Chicago, IL, USA). Potential target genes were identified by intersecting the prediction results from these tools. Subsequently, GO and KEGG pathway enrichment analyses were performed on candidate target genes to elucidate their potential functions and associated biological pathways. In both GO and KEGG analyses, a significance threshold of *p* < 0.05 was set to identify significantly enriched terms. Cytoscape software 3.7.1 (Cytoscape Consortium, San Diego, CA, USA) was used to construct miRNA-GO/KEGG-target gene networks, enabling visualization of relationships among miRNAs, their target genes, and associated pathways.

### 2.8. Validation of Target Relationships

**Figure 1:**
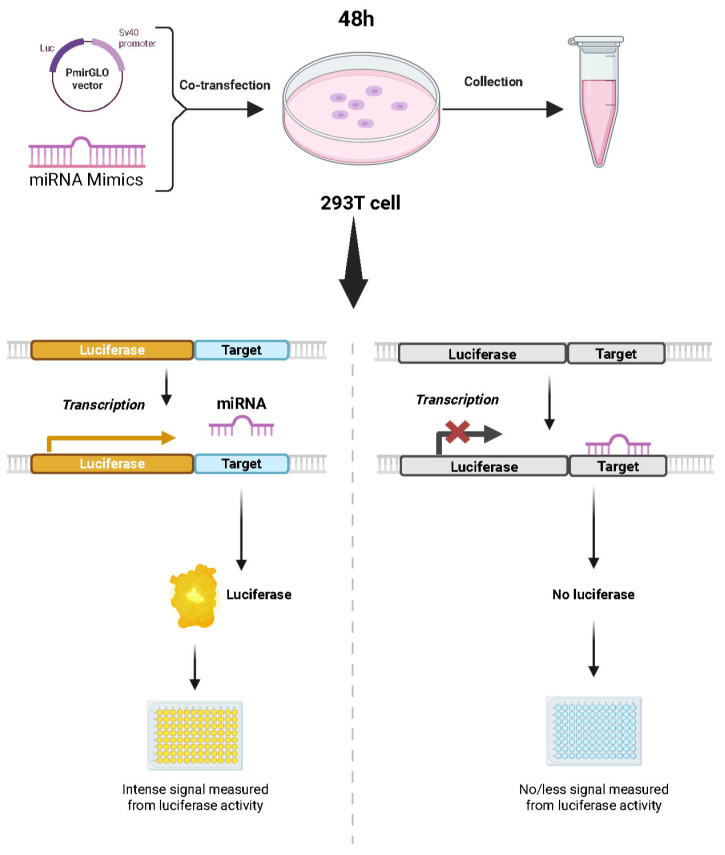
Diagram of the dual-luciferase reporter assay.

To validate the targeting relationships between miR-134-5p and miR-136-5p and their target genes, we used the dual-luciferase reporter assay (Figure 1). The backbone vector was amplified in LB broth (AMP+) at 37 °C for 12 h. The bacterial culture was processed to remove debris, and the vector was purified and digested with XhoI and XbaI (New England Biolabs, Ipswich, MA, USA). The vector was linearized via 1% agarose gel electrophoresis. Target binding sites for miR-134-5p and miR-136-5p within the target genes’ 3′UTR were identified using TargetScan, and primers were designed (Supplementary Table S1). The target fragments were synthesized, annealed into double-stranded DNA, and ligated into the linearized vector. The ligation product was transformed into competent cells, cultured, and plated on LB agar (AMP+). Selected colonies were cultured, and plasmid DNA was extracted and sequenced to verify the construct. The confirmed recombinant plasmid was propagated in E·coli for subsequent transfection experiments.

The 293T cells were transfected in four groups: miR-134/136-5p mimics or NC mimics (RiboBio, Guangzhou, China) with WT or MUT target gene vectors. Cells (2 × 10^5^ per well) were seeded in 24-well plates and transfected at ~95% confluence. Transfection complexes were formed by mixing Solution A (50 µL Opti-MEM (Thermo Fisher Scientific, Waltham, MA, USA) with 2 µL Lipofectamine 2000 (Invitrogen, Carlsbad, CA, USA)) and Solution B (50 µL Opti-MEM with 1.25 µL mimics/NC and 300 ng vector) at a 1:1 ratio, incubating this for 5 min at room temperature, and diluting it to 500 µL with Opti-MEM and adding this to the cells. After 6 h incubation at 37 °C with 5% CO_2_, the medium was replaced. For detection, cells were washed three times with 1× DPBS (Thermo Fisher Scientific, Waltham, MA, USA), lysed with 100 µL 1× PLB (Promega, Madison, WI, USA) for 15 min at 145 rpm, then centrifuged at 4 °C and 12,000 rpm for 30 s. Luciferase activity was measured by mixing 10 µL supernatant with 50 µL Luciferase Assay Reagent (Promega, Madison, WI, USA) to read RLU1, followed by adding 50 µL Stop Reagent (Promega, Madison, WI, USA) to read RLU2. The ratio (RLU1/RLU2) was recorded.

### 2.9. Morphological Observation

The skin tissues used in H&E staining experiments were obtained from fresh samples. Dorsal skin tissues were cut into 1 cm^2^ pieces, rinsed with PBS, and immediately immersed in 4% paraformaldehyde for 24 h for fixation. After fixation, the samples were dehydrated using a graded ethanol series, cleared in xylene, and embedded in paraffin. Thin sections were then prepared and stained with H&E. Morphological observations were conducted using a microscope.

### 2.10. Immunofluorescence Staining

The skin tissues used in the immunofluorescence staining experiments were also obtained from fresh samples, which were rinsed with PBS and fixed in paraformaldehyde for 24 h before experimentation. Tissue sections were dewaxed in xylene and rehydrated using a graded ethanol series. These sections were subjected to heat-induced epitope retrieval in 0.01 M sodium citrate buffer to expose antigens. After antigen retrieval, the sections were blocked with blocking solution at room temperature for 1 h and then incubated with primary antibodies overnight at 4 °C. The primary antibodies used were anti-BCL2 (Bioss, Beijing, China, bsm-1174R, dilution 1:400), anti-KI67 (Bioss, Beijing, China, bsm-52455R, dilution 1:400), anti-VEZF1 (Bioss, Beijing, China, bs-12791R, dilution 1:400), and anti-MAP3K1 (Proteintech, Rosemont, IL, USA, 19970-1-AP, dilution 1:400). After the incubation period, sections were uniformly covered with the secondary antibody and incubated for 1 h in the dark, followed by three washes with PBS. Nuclei were counterstained with DAPI (Sigma, Saint Louis, MO, USA, D8417, dilution 1:1000) for 8–10 min at room temperature in the dark, with three additional washes with PBS. These sections were then mounted and examined to assess BCL2, KI67, MAP3K1, and VEZF1 expression in various hair follicle structures.

### 2.11. Statistical Analysis

Each experiment was conducted independently in triplicate. Data analysis was performed using the GraphPad Prism 8 software (GraphPad Software, San Diego, CA, USA). Student’s *t*-test was used to compare differences between the two groups. When evaluating differences among multiple groups, one-way analysis of variance (ANOVA) was performed. A *p*-value of <0.05 was considered statistically significant.

## 3. Results

### 3.1. Fgf21 Knockout Delays the Transition from Telogen to Anagen in Hair Follicles

**Figure 2:**
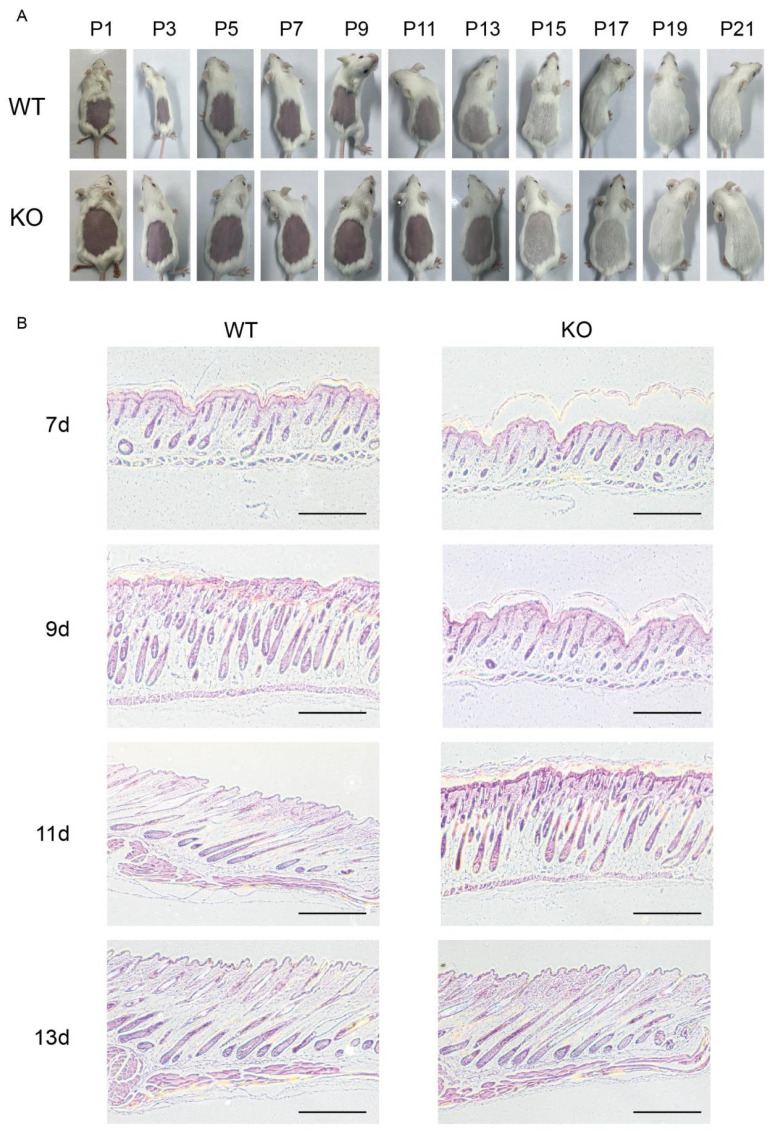
Effects of *Fgf21* knockout on hair follicle cycle transition. (**A**) Time-series images showing the dorsal skin and hair development in WT and *Fgf21^−^/^−^* mice from day 1 (P1) to 21 (P21) post-depilation. (**B**) H&E staining illustrating hair follicle structures in WT and *Fgf21^−^/^−^* mice on day 7 (7 d); day 9 (9 d); day 11 (11 d); and day 13 (13 d) post-depilation. Scale bar = 1000 μm.

During telogen, the skin of friend virus B-type (FVB) mice typically appears pink, gradually turns pale pink, and begins to grow white hair at the onset of anagen. To investigate the role of *Fgf21* in hair follicle cycle transition, we synchronized the hair follicle cycles of 8-week-old *Fgf21^−^/^−^* and WT mice through depilation. Immediately after depilation, the skin of all the mice appeared pink, indicating that they were in the telogen phase, with no significant differences observed between the two groups. On day 7 post-depilation, both *Fgf21^−^/^−^* and WT mice retained pink skin, confirming that they remained in the telogen phase. On day 9, while the skin of *Fgf21^−^/^−^* mice remained pink, that of WT mice began to turn pale pink, indicating the initial entry into the anagen phase. By day 11, *Fgf21^−^/^−^* mice showed no significant changes, whereas WT mice began to grow fine vellus hair. On day 13, *Fgf21^−^/^−^* mice gradually turned pale pink, marking the initial entry into the anagen phase, whereas WT mice had already developed longer hair covering the skin (Figure 2A).

To further investigate the impact of *Fgf21* knockout on hair follicle cycle transition, we performed hematoxylin and eosin (H&E) staining on depilated skin tissues of *Fgf21^−^/^−^* and WT mice. The results were consistent with phenotypic observations. On day 7 post-depilation, both groups exhibited typical in the telogen phase features with shortened hair follicles and tightly packed cell layers between the inner and outer root sheaths, showing no significant differences. By day 9, WT mice began to show increased hair follicle length, enlarged dermal papilla, and the thickened dermis, indicating a transition to anagen, whereas *Fgf21^−^/^−^* mice remained in telogen with shorter hair follicles and shrunken dermal papilla. By day 11, most hair follicles in WT mice had extended downward close to the adipose layer, with increased dermal thickness and dermal papilla enlargement, confirming their entry into the anagen phase. In contrast, *Fgf21^−^/^−^* mice only began to extend their follicles into the dermis and indicated a transition to the anagen phase. By day 13, both groups exhibited increased follicle length and dermal thickening, confirming their eventual progression into the anagen phase. These results indicate that *Fgf21* knockout delays the transition from telogen to anagen in hair follicles (Figure 2B).

### 3.2. Expression Analysis and Functional Annotation of miRNAs

**Figure 3:**
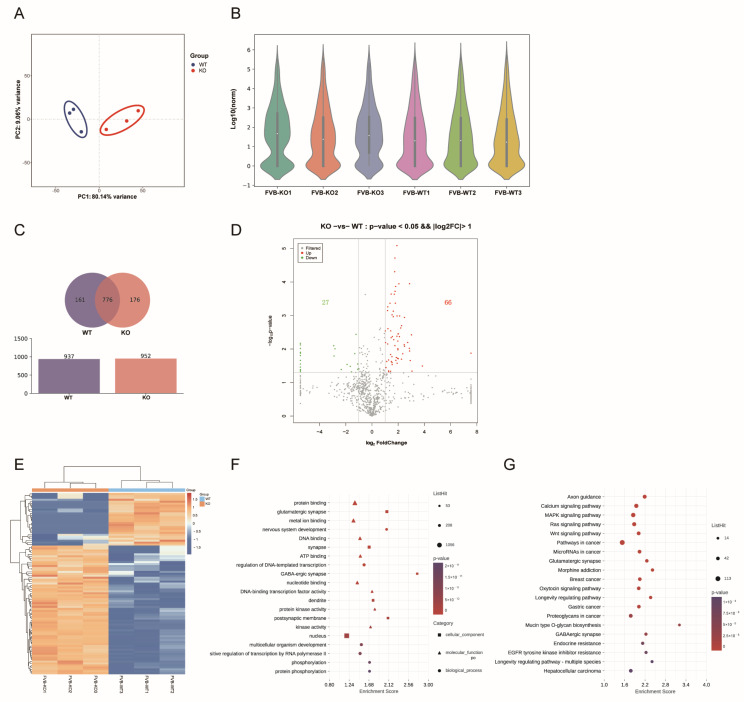
Expression analysis and functional annotation of miRNAs. (**A**) PCA plot of the distribution of miRNAs expression in dorsal skin tissues of WT and *Fgf21^−^/^−^* mice. (**B**) Violin plots of the distribution of miRNAs expression in six different samples of WT and *Fgf21^−^/^−^* mice. (**C**) Venn diagram comparing miRNAs expression between WT and *Fgf21^−^/^−^* mice. A total of 776 miRNAs were commonly expressed between the two groups, with 176 miRNAs specifically expressed in *Fgf21^−^/^−^* mice and 161 miRNAs specifically expressed in WT mice. (**D**) Volcano plot showing the differentially expressed miRNAs between the two groups. The plot highlights the 66 upregulated and 27 downregulated miRNAs. (**E**) Hierarchical clustering heatmap of DEmiRNAs, showing the expression patterns of differentially expressed miRNAs in the dorsal skin tissues of WT and *Fgf21^−^/^−^* mice. Color gradations indicate relative changes in miRNAs expression levels, with yellow indicating upregulation and blue indicating downregulation. (**F**) GO functional annotation of target genes of DEmiRNAs. The bubble chart displays the top 20 most significantly enriched GO terms. (**G**) KEGG pathway enrichment analysis of target genes of DEmiRNAs. The bubble chart displays the top 20 most significantly enriched KEGG pathways.

To investigate the impact of *Fgf21* knockout on hair follicle growth and development, we performed miRNA sequencing of the dorsal skin tissues of *Fgf21^−^/^−^* and WT mice, each with three biological replicates per group. After quality control and sequence alignment, we identified 937 miRNAs in the WT group and 952 miRNAs in the *Fgf21^−^/^−^* group. Principal component analysis (PCA) revealed distinct differences in miRNA expression between the experimental and control groups, whereas the intra-group samples exhibited high reproducibility, indicating the consistency of experimental operations and reliability of the results (Figure 3A). Violin plots also illustrated the distribution of miRNA expression levels in each sample (Figure 3B).

A Venn diagram analysis of miRNAs from *Fgf21^−^/^−^* and WT mice revealed that 776 miRNAs were commonly expressed between the two groups, 176 miRNAs were specifically expressed in the *Fgf21^−^/^−^* group, and 161 miRNAs were specifically expressed in the WT group (Figure 3C). A comparative analysis of expression levels between the two groups identified 93 differentially expressed miRNAs, of which 66 were upregulated and 27 were downregulated. Volcano plots and hierarchical clustering heat maps also showed the distribution and clustering of such differentially expressed miRNAs (Figure 3D,E).

To further elucidate the functions of these differentially expressed miRNAs (DEmiRNAs), we performed Gene Ontology (GO) and Kyoto Encyclopedia of Genes and Genomes (KEGG) functional annotation and enrichment analysis of their target gene sets using miRanda 2.066 prediction software. GO analysis revealed that the host genes of the DEmiRNAs were primarily enriched in functions such as protein binding, DNA binding, nucleotide binding, multicellular organismal development, positive regulation of RNA polymerase II transcription, and protein phosphorylation (Figure 3F). KEGG analysis revealed that the target genes of DEmiRNAs were associated with several key signaling pathways during hair follicle development, including the MAPK signaling pathway, Ras signaling pathway, and Wnt signaling pathway (Figure 3G). These results suggest that miRNAs are likely involved in the molecular pathways of hair follicle development by regulating their target gene expression.

### 3.3. Clustering Analysis and Expression Level Detection of Hair Follicle-Related miRNAs

**Figure 4:**
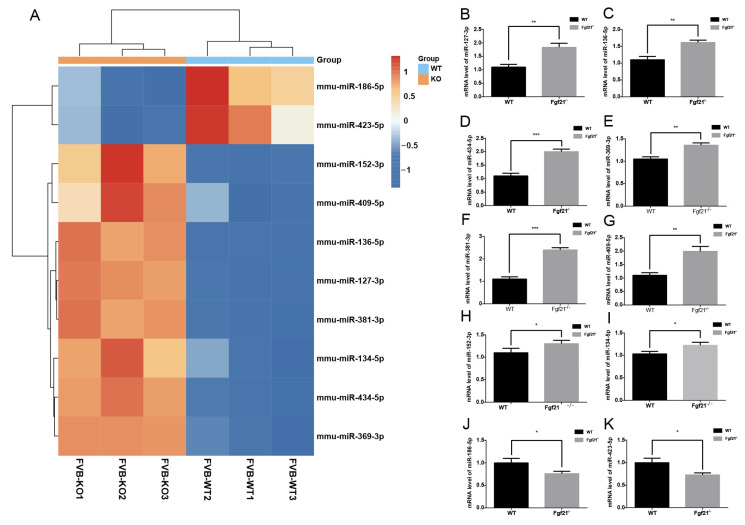
Effects of *Fgf21* knockout on the expression of hair follicle-related miRNAs and validation. (**A**) Hierarchical clustering heatmap of the expression patterns of 10 hair follicle-related miRNAs in the dorsal skin tissues of WT and *Fgf21^−^/^−^* mice. Color changes denote relative changes in miRNA expression levels, with upregulation indicated by red and downregulation indicated by blue. (**B**–**K**) RT-qPCR results indicating the relative expression levels of specific miRNAs in the dorsal skin tissues of WT and *Fgf21^−^/^−^* mice. RT-qPCR results were consistent with the sequencing data trends. * *p* < 0.05, ** *p* < 0.01, *** *p* < 0.005. Each group included three mice, and experiments were repeated three times independently.

To further explore the effects of *Fgf21* knockout on the expression of hair follicle-related miRNAs, we identified 10 miRNAs associated with hair follicle growth and development and performed clustering analysis on their expression levels (Figure 4A). The clustering heatmap revealed distinct miRNAs expression patterns between the two groups. Specifically, miR-423-5p and miR-186-5p were downregulated in *Fgf21^−^/^−^* mice, whereas miR-409-5p, miR-127-3p, miR-134-5p, miR-381-3p, miR-152-3p, miR-434-5p, miR-369-3p, and miR-136-5p were upregulated. These results indicate that there are significant differences in miRNA expression levels between the dorsal skin tissues of WT and *Fgf21^−^/^−^* mice.

To validate the accuracy of the miRNA sequencing results, we performed RT-qPCR analysis and selected miRNAs using U6 as an endogenous reference miRNA. The RT-qPCR results showed that the expression levels of miR-409-5p, miR-127-3p, miR-134-5p, miR-381-3p, miR-152-3p, miR-434-5p, miR-369-3p, and miR-136-5p were upregulated in *Fgf21^−^/^−^* mice, whereas those of miR-186-5p and miR-423-5p were downregulated (Figure 4B–K). These findings are consistent with the trends observed in the sequencing data. These results further confirmed the role of *Fgf21* in modulating the expression of hair follicle-related miRNAs.

### 3.4. Clustering Analysis and Network Construction of Target Genes

**Figure 5:**
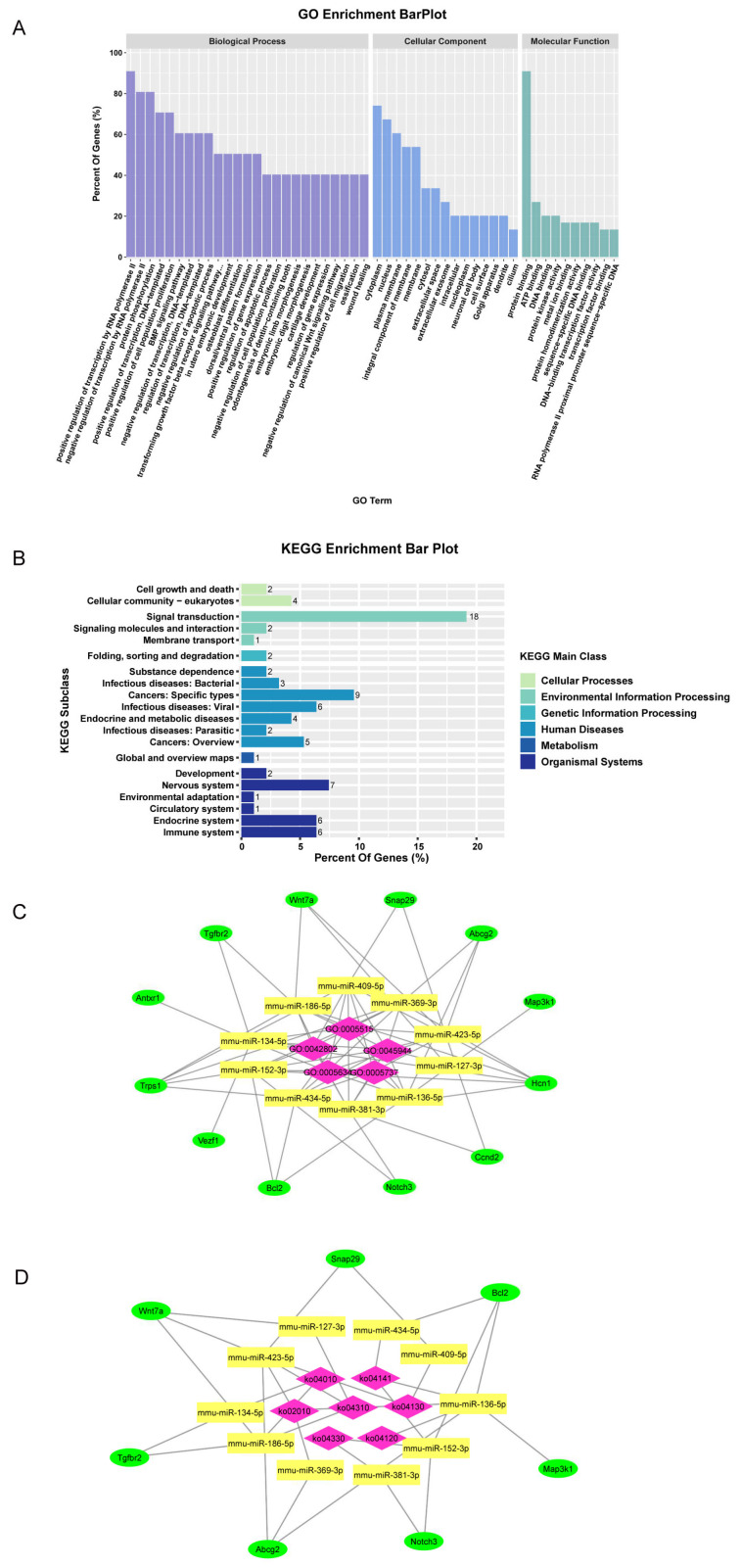
Clustering analysis and network construction of target genes. (**A**) GO functional analysis of target genes. (**B**) KEGG pathway enrichment of target genes. (**C**) miRNAs–GO–target gene network showing interactions between miRNAs and their target genes within GO terms. Green nodes represent target genes, purple nodes represent GO terms, and yellow nodes represent miRNAs. Lines indicate regulatory relationships between miRNAs and target genes, as well as associations between target genes and GO terms. (**D**) miRNAs–KEGG–target gene network showing interactions between miRNAs and their target genes within KEGG pathways. Green nodes represent target genes, purple nodes represent KEGG pathways, and yellow nodes represent miRNAs. Lines indicate regulatory relationships between miRNAs and target genes, as well as associations between target genes and KEGG pathways.

To elucidate the molecular mechanisms by which *Fgf21* knockout influences hair follicle growth and development, we identified target genes for the 10 hair follicle-related miRNAs and performed GO and KEGG functional enrichment analysis. GO functional enrichment analysis revealed that the target genes are primarily involved in biological processes including regulation of transcription from the RNA polymerase II promoter, protein phosphorylation, and regulation of transcription from the DNA template. Subcellular localization analysis showed that the target genes were predominantly located in the cytoplasm, nucleus, and plasma membrane. In terms of molecular function, the target genes were primarily involved in binding to proteins, ATP, and DNA, as well as with protease activation (Figure 5A). KEGG pathway enrichment analysis indicated that the target genes were enriched in pathways related to signal transduction, cancer, and nervous, endocrine, and immune systems (Figure 5B).

By combining literature reports, we selected hair follicle-related target genes for GO and KEGG enrichment analyses. GO analysis revealed that these target genes are primarily enriched in biological processes such as protein binding, homologous protein binding, and the regulation of transcription from RNA polymerase II promoter, as well as in cellular components like the cytoplasm and nucleus. Consequently, we constructed a miRNAs–GO–target gene network association diagram (Figure 5C). KEGG analysis showed that these target genes were significantly enriched in hair follicle development-related pathways, including the Wnt signaling pathway, SNARE interactions in vesicular transport, ABC transporters, protein processing in the endoplasmic reticulum, the Notch signaling pathway, ubiquitin-mediated proteolysis, and the MAPK signaling pathway. We also constructed a miRNAs–KEGG–target gene network association diagram (Figure 5D). These network diagrams highlight the potential molecular processes through which *Fgf21* may regulate hair follicle growth and development through miRNAs, providing new insights into the molecular basis of this process. Thus, miRNAs may serve as a potential therapeutic target for follicle-related diseases.

### 3.5. Functional Implications of miRNAs in Hair Follicle Development

**Figure 6:**
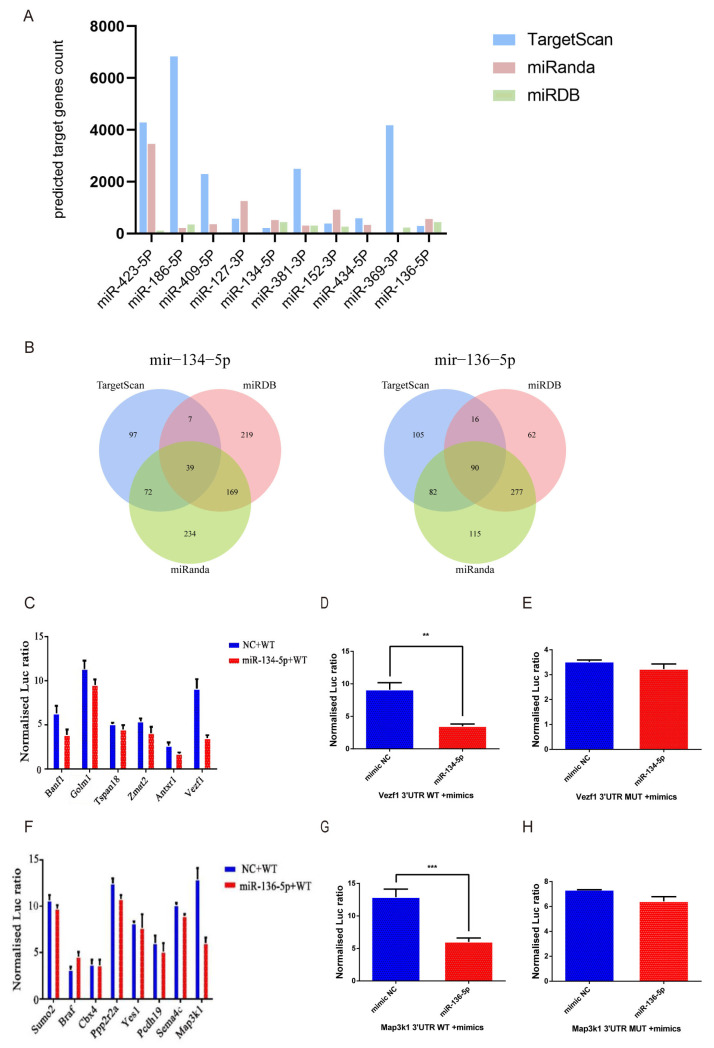
Target gene prediction and dual-luciferase reporter assay. (**A**) Bar chart shows the number of target genes for 10 miRNAs predicted by TargetScan, miRanda, and miRDB. (**B**) Venn diagram showing the predicted target genes of miR-134-5p and miR-136-5p, intersected from TargetScan, miRanda, and miRDB. Numbers indicate the number of target genes predicted by each software and their overlaps. (**C**) Bar chart showing the dual-luciferase reporter assay results for miR-134-5p and its potential target genes *Banf1*, *Golm1*, *Tspan18*, *Zmat2*, *Antxr1*, and *Vezf1*. Blue bars represent the NC mimics group and red bars represent the miR-134-5p mimics group. (**D**) Bar chart showing the dual-luciferase reporter assay results for *Vezf1* 3′UTR WT co-transfected with miR-134-5p mimics and *Vezf1* 3′UTR WT co-transfected with NC mimics. Blue bars represent the NC mimics group and red bars represent the miR-134-5p mimics group. (**E**) Bar chart showing the dual-luciferase reporter assay results for *Vezf1* 3′UTR MUT co-transfected with miR-134-5p mimics and *Vezf1* 3′UTR MUT co-transfected with NC mimics. Blue bars represent the NC mimics group and red bars represent the miR-134-5p mimics group. (**F**) Bar chart showing the dual-luciferase reporter assay results for miR-136-5p and its potential target genes *Sumo2*, *Braf*, *Cbx4*, *Ppp2r2a*, *Yes1*, *Pcdh19*, *Sema4c*, and *Map3k1*. Blue bars represent the NC mimics group and red bars represent the miR-136-5p mimics group. (**G**) Bar chart showing the dual-luciferase reporter assay results for *Map3k1* 3′UTR WT co-transfected with miR-136-5p mimics and *Map3k1* 3′UTR WT co-transfected with NC mimics. Blue bars represent the NC mimics group and red bars represent the miR-136-5p mimics group. (**H**) Bar chart showing the dual-luciferase reporter assay results for *Map3k1* 3′UTR MUT co-transfected with miR-136-5p mimics and *Map3k1* 3′UTR MUT co-transfected with NC mimics. Blue bars represent the NC mimics group and red bars represent the miR-136-5p mimics group. ** *p* < 0.01, *** *p* < 0.005. Each experiment was independently repeated three times.

To identify the potential target genes influenced by *Fgf21* knockout, we used three prediction tools—TargetScan, miRanda, and miRDB—to predict target genes for the 10 hair follicle-related miRNAs (Figure 6A). By comparing the prediction results from these tools, we identified miR-134-5p and miR-136-5p as the miRNAs with the highest number of predicted target genes among the three software programs. Specifically, miR-134-5p had 39 predicted target genes, while miR-136-5p had 90. To further validate these findings, we constructed Venn diagrams to visualize the intersection of these target gene predictions for further experimental analysis (Figure 6B).

For miR-134-5p, we selected six high-scoring target genes associated with hair follicle development from the 39 predicted targets for dual-luciferase reporter assays. The results showed that *Banf1*, *Golm1*, *Tspan18*, *Zmat2*, and *Antxr1* did not significantly interact with miR-134-5p (Figure 6C). However, *Vezf1* showed a significant reduction in luciferase activity when co-transfected with miR-134-5p mimics compared to the negative control (NC) mimics (Figure 6D). Mutation assays further confirmed that *Vezf1* is a target gene of miR-134-5p, as the mutant *Vezf1* vector showed no significant changes in luciferase activity when co-transfected with miR-134-5p mimics and NC mimics (Figure 6E).

For miR-136-5p, we selected eight target genes that not only scored highly in the bioinformatics prediction but also have relevance to hair follicle growth. The results showed that *Sumo2*, *Braf*, *Cbx4*, *Ppp2r2a*, *Yes1*, *Pcdh19*, and *Sema4c* did not show significant interactions with miR-136-5p (Figure 6F). In contrast, *Map3k1* exhibited a significant reduction in luciferase activity when co-transfected with miR-136-5p mimics compared to NC mimics (Figure 6G). Mutation assays further confirmed that *Map3k1* is a target gene of miR-136-5p, as the mutant *Map3k1* vector did not show significant changes in luciferase activity when co-transfected with miR-136-5p mimics and NC mimics (Figure 6H).

### 3.6. Fgf21 Regulates Expression and Localization of Vezf1 and Map3k1 in the Hair Follicle Growth Cycle

**Figure 7:**
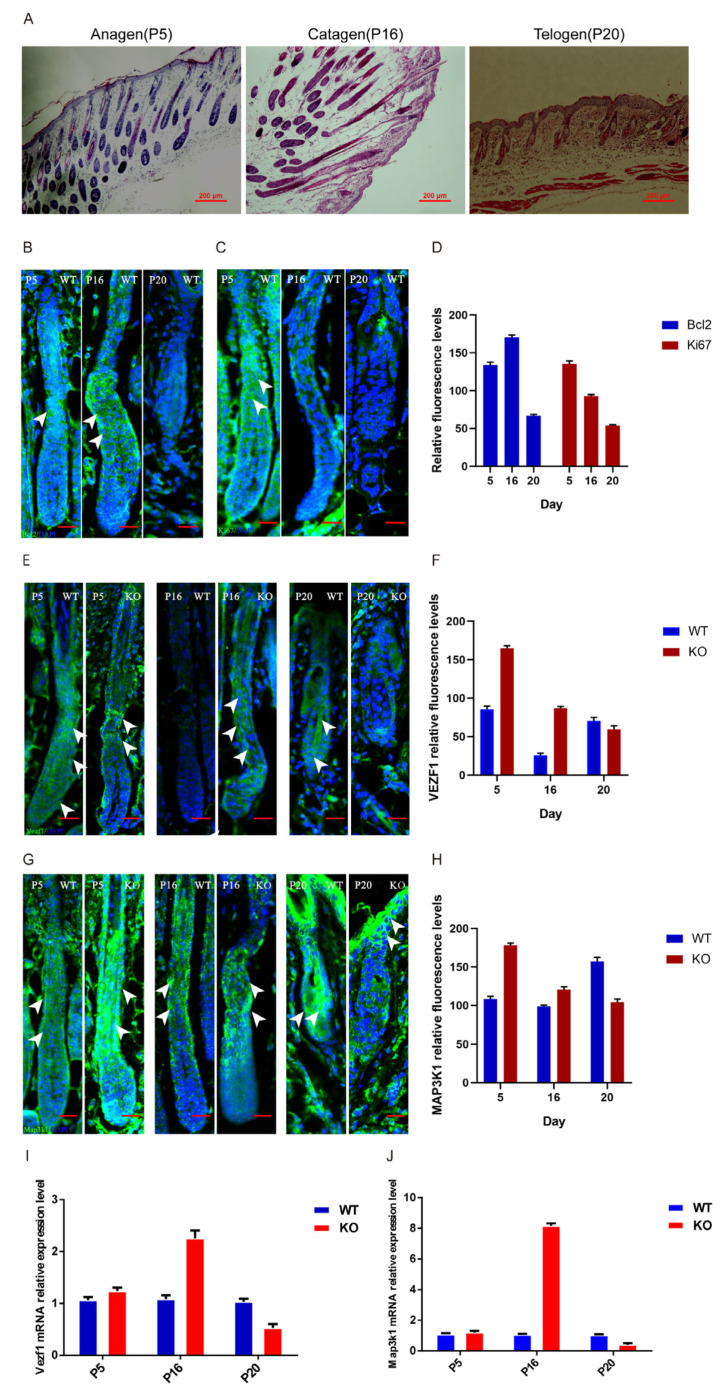
Identification of the hair follicle growth cycle and the effects of *Fgf21* on target gene expression. (**A**) Histological staining (H&E) showing the hair follicle structures of dorsal skin from P5, P16, and P20 mice. Scale bar = 200 μm. (**B**) Immunofluorescence staining showing the localization of BCL2 (green) in hair follicles of P5, P16, and P20 mice, with DAPI (blue) used for nuclear staining. Scale bar = 100 μm. (**C**) Immunofluorescence staining showing the localization of Ki67 (green) in hair follicles of P5, P16, and P20 mice, with DAPI (blue) used for nuclear staining. Scale bar = 100 μm. (**D**) Bar chart showing the quantification of BCL2 and KI67 fluorescence intensity in WT mice at P5, P16, and P20. (**E**) Immunofluorescence staining showing the localization of VEZF1 (green) in hair follicles of P5, P16, and P20 WT and *Fgf21^−^/^−^* mice, with DAPI (blue) used for nuclear staining. Scale bar = 100 μm. (**F**) Bar chart showing the quantification of VEZF1 fluorescence intensity in WT and *Fgf21^−^/^−^* mice at P5, P16, and P20. (**G**) Immunofluorescence staining showing the expression of MAP3K1 (green) in hair follicles of P5, P16, and P20 WT and *Fgf21^−^/^−^* mice, with DAPI (blue) used for nuclear staining. Scale bar = 100 μm. (**H**) Bar chart showing the quantification of MAP3K1 fluorescence intensity in WT and *Fgf21^−^/^−^* mice at P5, P16, and P20. (**I**) RT-qPCR analysis of Vezf1 expression in dorsal skin tissues from P5, P16, and P20 mice. (**J**) RT-qPCR analysis of Map3k1 expression in dorsal skin tissues from P5, P16, and P20 mice. P5, P16, and P20 correspond to postnatal days 5, 16, and 20 in mice, representing the anagen phase, catagen phase, and telogen phase of the hair follicle cycle, respectively. White arrows have been included to highlight protein expression sites throughout the hair follicle cycle. Each group included three mice, and the experiments were independently repeated three times.

To characterize the hair follicle growth cycle, we examined the dorsal skin tissues of mice on postnatal day 5 (P5), 16 (P16), and 20 (P20). We observed the distribution of hair follicles with enlarged dermal papilla regions at P5 within the dermis and subcutaneous tissue, which is a feature of the anagen phase. At P16, the hair follicles extended through the epidermis, dermis, and subcutaneous tissue, with hair shafts reaching the epidermal surface, indicative of the catagen phase. Hair follicles were reduced in size and in a resting state at P20, which is a characteristic of the telogen phase. Therefore, we, respectively, define P5, P16, and P20 as the anagen, catagen, and telogen phases. These observations were consistent with the findings of Müller-Röver et al. [21] (Figure 7A).

Immunofluorescence staining was performed to detect the expression of the apoptosis marker BCL2 and the proliferation marker KI67, with quantification via ImageJ 1.45. During the anagen phase, KI67 exhibited the highest expression levels in hair follicle stem cells and throughout the hair follicle, while BCL2 was significantly expressed in the outer root sheath. In the catagen phase, KI67 expression was attenuated, and BCL2 expression was highest in both the outer and inner root sheaths. During the telogen phase, the expression levels of both KI67 and BCL2 were low (Figure 7B–D).

The expression levels and protein localization of *Vezf1* and *Map3k1* during the natural growth cycle of hair follicles in WT and *Fgf21^−^/^−^* mice were examined using immunofluorescence staining and quantified via Image J. During the anagen phase, VEZF1 and MAP3K1 were expressed in the outer root sheath and inner root sheath of hair follicles. During the catagen phase, VEZF1 was expressed in the hair shaft, inner root sheath, and outer root sheath of hair follicles in *Fgf21^−^/^−^* mice, with higher expression levels than WT mice. MAP3K1 was expressed in the outer and inner root sheaths of both WT and *Fgf21^−^/^−^* mice. During the telogen phase, VEZF1 was predominantly expressed in the hair shaft of WT mice, whereas MAP3K1 was mainly expressed in the outer root sheath and the hair shaft of WT mice (Figure 7E–H).

RT-qPCR analysis revealed that during the anagen phase, *Vezf1* and *Map3k1* expression levels were not significantly different between WT and *Fgf21^−^/^−^* mice (*p* > 0.05). However, in the catagen phase, *Vezf1* expression in *Fgf21^−^/^−^* mice was 2.27 times higher than in WT mice, while *Map3k1* expression level was 8.3 times higher (*p* < 0.05). During the telogen phase, *Vezf1* expression levels in *Fgf21^−^/^−^* mice were 0.51 times lower than those in WT mice (Figure 7I,J), while the *Map3k1* expression level was 0.5 times lower (*p* < 0.005). These results indicate that *Fgf21* knockout alters the expression patterns, including the localization and expression levels, of VEZF1 and MAP3K1 during the hair follicle growth cycle, suggesting that *Fgf21* may regulate hair follicle growth and cycle transition by modulating the expression of these genes.

## 4. Discussion

The hair follicle growth cycle, comprising the anagen, catagen, and telogen phases, is a complex and dynamic physiological process regulated by multiple signaling pathways. The FGF family plays crucial roles in the regulation of hair follicle growth and development. Specifically, the interaction between FGF and Wnt signaling pathways in the dermal papilla regulates the duration of the anagen phase through positive and negative feedback loops [12]. *Fgf7*, secreted by dermal papilla cells (DPCs), promotes the proliferation of DPCs and hair follicle stem cells via the Wnt signaling pathway and induces hair follicle stem cell (HFSC) differentiation [22]. Additionally, *Fgf1* and *Fgf2* promote hair growth, while *Fgf5* induces catagen formation and reduces hair length. *Fgf12* is expressed in the outer root sheath cells during anagen, and its knockdown delays the transition from telogen to anagen in mice and reduces the length of whisker hair follicle, whereas overexpression of *Fgf12* increases the migration of the outer root sheath cells [23]. *Fgf21*, a relatively new member of the FGF family, produces a secreted protein [24]; however, its role in the hair follicle growth cycle has not yet been fully explored. In this study, we generated a *Fgf21^−^/^−^* mouse model using the CRISPR/Cas9 system and performed high-throughput miRNA sequencing on the dorsal skin tissues of WT and *Fgf21^−^/^−^* mice, identifying 93 differentially expressed miRNAs. We further selected 10 hair follicle-related miRNAs (miR-409-5p, miR-127-3p, miR-134-5p, miR-381-3p, miR-152-3p, miR-434-5p, miR-369-3p, miR-136-5p, miR-186-5p, and miR-423-5p) and validated their expression using RT-qPCR; the results were consistent with the sequencing data. Through synchronized hair follicle cycle experiments, we observed a delayed transition from telogen to anagen in *Fgf21^−^/^−^* mice, indicating a crucial role for *Fgf21* in the regulation of the hair follicle growth cycle. Liu et al. demonstrated that, compared to WT mice, *Fgf21^−^/^−^* mice exhibit a slower hair regrowth rate, a significant reduction in hair shaft density, and markedly decreased expression levels of *Erk* and *Akt* [25]. Our findings reveal the role of *Fgf21* in the hair follicle growth cycle, though the specific mechanisms require further investigation.

*Vezf1*, a zinc-finger transcription factor, is specifically expressed in vascular endothelial cells and plays a critical role in vascular development [26]. Liang et al. demonstrated that reduced expression of *Vezf1* can disrupt the expression of genes related to vascular endothelial cells in human induced pluripotent stem cells (iPSCs) [27]. Consequently, iPSCs regulated by miR-495 exhibited upregulated *Vezf1* expression, thereby promoting the proliferation of vascular endothelial cells [27]. Kuschnerus et al. found that miR-483-3p reduces *Vezf1* expression, leading to increased apoptosis of vascular endothelial cells and diminished regenerative capacity mediated by M2 macrophages [28]. These findings suggest that *Vezf1* may be regulated by multiple miRNAs, forming a complex regulatory network. In this study, we identified a targeting relationship between miR-134-5p and *Vezf1*, suggesting that miR-134-5p may influence *Vezf1* expression in a manner distinct from miR-495 and miR-483-3p, thereby affecting the proliferation and regenerative capacity of vascular endothelial cells. *Fgf21* regulates *Vezf1* expression during the hair follicle cycle. Research shows *Vezf1* can indirectly influence the VEGF pathway by regulating VEGF receptor expression. For example, *Vezf1*, with *Etv2*, activates *Flt1* transcription [29]. *Flt1*, a major VEGF receptor, when upregulated, boosts VEGF signaling, promoting angiogenesis [30]. Vascular endothelial growth factor (VEGF), also known as the “vascular permeability factor”, is overexpressed in the outer root sheath keratinocytes of hair follicles, promoting the formation of perifollicular vasculature and enhancing hair regeneration [31]. Our study shows that *Fgf21* plays a key role in regulating the hair follicle cycle. It likely modulates metabolic and proliferative processes via the FGF family, suggesting it may share functional similarities with VEGF in promoting hair follicle development. We observed that VEZF1 protein was present in the outer root sheath of WT mice during telogen, but absent in *Fgf21^−^/^−^* mice. Since *Vezf1* is essential for vascular development in mice [32], it may function similarly to VEGF by enhancing blood supply, thereby promoting hair follicle growth and development. Miyashita et al. showed that *Vezf1* regulates the proliferation, formation, and migration of endothelial cells during vascular development [33]. Hair follicles, as skin appendages, rely on the vascular network of the dermal papilla to supply nutrients. In this study, we found that VEZF1 was expressed in the dermal papilla of WT mice during anagen and telogen, whereas no expression was detected in *Fgf21^−^/^−^* mice. This suggests that *Fgf21* deficiency may lead to the absence of *Vezf1*, thereby affecting proliferation and reconstruction of the vascular network in the dermal papilla. This, in turn, reduces the nutrient supply from the blood to the hair follicle, delaying the transition of hair follicles from telogen to anagen.

*Map3k1*, a member of the mitogen-activated protein kinase kinase kinase (MAP3K) family, plays a crucial role in cell proliferation and apoptosis. Research by Andrew Parker et al. has shown that *Map3k1* regulates keratinocyte migration and actin stress fiber formation via the JNK signaling pathway, while also independently modulating cell proliferation and apoptosis through this pathway [34]. In our study, we observed a significant accumulation of MAP3K1 protein in the outer and inner root sheaths of hair follicles during the catagen phase in both WT and *Fgf21^−^/^−^* mice. Based on Parker’s findings, we speculated that *Map3k1* regulates apoptosis in hair follicle cells via the JNK signaling pathway during catagen. Epithelial–mesenchymal transition (EMT) is a key cellular process. Chang et al. demonstrated that *Map3k1* is a target gene of miR-145-5p and that its overexpression negatively regulates miR-145-5p expression through the JNK signaling pathway, thereby inhibiting EMT in non-small cell lung cancer cells [35]. In this study, we validated the targeting relationship between miR-136-5p and *Map3k1*, suggesting that miR-136-5p may negatively regulate *Map3k1* through the JNK signaling pathway, thereby affecting hair follicle growth and development. But it is uncertain whether this process depends on the regulation of the *Fgf21*. Sennett et al. demonstrated that the EMT between dermal cells and epithelial stem cells influences hair follicle induction and formation [1]. During the anagen phase of the hair follicle growth cycle, the interaction between mesenchymal dermal papilla cells and epithelial matrix cells induces hair shaft formation [1]. The hair shaft is formed by rapidly proliferating and differentiating matrix cells that further differentiate and migrate to form the inner root sheath, thereby determining the shape of the hair [36]. In our study, the MAP3K1 protein was detected in the hair bulb, outer root sheath, and inner root sheath of hair follicles during the anagen phase in both WT and *Fgf21^−^/^−^* mice. However, during telogen, MAP3K1 expression levels were reduced in *Fgf21^−^/^−^* mice. This suggests that *Fgf21* deficiency may lead to decreased *Map3k1* expression, thereby affecting the differentiation and migration of matrix cells and ultimately influencing the transition from telogen to anagen phase in hair follicles.

In summary, our data show that *Fgf21* gene regulation leads to significant changes in the expression levels and protein localization of its target genes, *Vezf1* and *Map3k1*, within hair follicles. However, the exact mechanisms by which *Vezf1* and *Map3k1* regulate hair follicle development are still unknown. Consequently, our next study will focus on investigating the specific functions of *Vezf1* and *Map3k1* in hair follicles.

## 5. Conclusions

**Figure 8:**
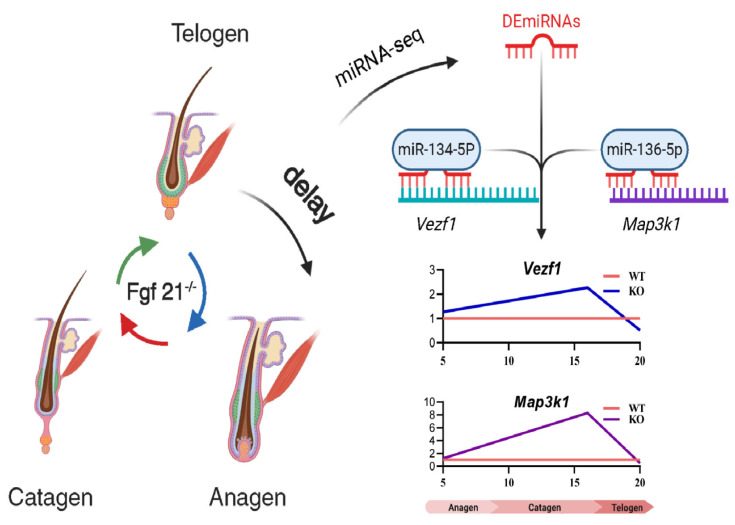
Model for regulation of hair follicle cycle by *Fgf21*-related miRNAs.

*Fgf21* knockout delays the transition from telogen to anagen in hair follicles. Dual-luciferase assays confirm that miR-134-5p directly targets *Vezf1* and miR-136-5p targets *Map3k1.* We found that the expression levels and protein localization of the target genes *Vezf1* and *Map3k1* in hair follicles were significantly altered by *Fgf21* treatment. The findings demonstrate that *Fgf21* modulates miRNA expression profiles to regulate hair follicle development, providing theoretical insights for hair follicle biology research and the clinical management of hair growth disorders (Figure 8).

## Data Availability

The original contributions presented in this study are included in the article/Supplementary Materials. Further inquiries can be directed to the corresponding author.

## Supplementary Materials

The following supporting information can be downloaded at: https://www.mdpi.com/article/10.3390/biology14050526/s1, Figure S1: The Genotype identification of WT and *Fgf21^−^/^−^* mice results. The band labeled “^−/−^” represents the *Fgf21* knock out mice (634 bp). “WT” represents wild-type mice (743 bp). Experiments were repeated three times, and the results were consistent; Table S1: The information on the sequences of the primers used in this study.

## Abbreviations

The following abbreviations are used in this manuscript:

ANOVA	one-way analysis of variance
DPC	dermal papilla cell
EMT	epithelial–mesenchymal transition
FGF	fibroblast growth factor
*Fgf21*	fibroblast growth factor 21
FV1b	friend virus susceptibility 1b
FVB	friend virus B-type
GO	gene ontology
H&E	hematoxylin and eosin
HFSC	hair follicle stem cell
iPSC	induced pluripotent stem cell
KEGG	Kyoto Encyclopedia of Genes and Genomes
Map3k1	mitogen-activated protein kinase kinase kinase 1
miRNA	microRNA
NC	negative control
PCA	principal component analysis
RT-qPCR	real-time quantitative PCR
shh	sonic hedgehog
TPM	transcripts per million
VEGF	vascular endothelial growth factor
Vezf1	vascular endothelial zinc finger 1
WT	wild-type
