# Supplementary material for: Fgf21 Deficiency Delays Hair Follicle Cycling and Modulates miRNA–Target Gene Interactions in Mice
Source: Biology (Basel). 2025 May 9;14(5):526. doi: 10.3390/biology14050526 (PMC12109541; doi:10.3390/biology14050526)
Supplement: Supplementary file 1 [file biology-14-00526-s001.zip › Figure S1.pdf]

A

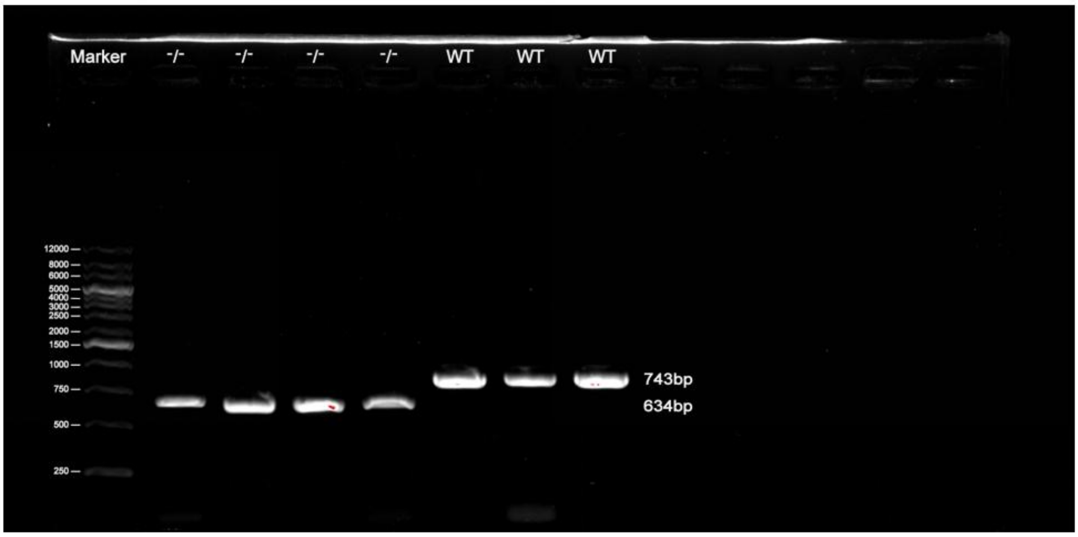

Figure S1. The Genotype identification of WT and Fgf21-/- mice results.The band labeled “-/-” represents the Fgf21 knock out mice(634bp). “WT” represents wild-type mice(743bp). Experiments were repeated three times, and the results were consistent.
