# Supplementary material for: Fgf21 Deficiency Delays Hair Follicle Cycling and Modulates miRNA–Target Gene Interactions in Mice
Source: Biology (Basel). 2025 May 9;14(5):526. doi: 10.3390/biology14050526 (PMC12109541; doi:10.3390/biology14050526)
Supplement: Supplementary file 1 [file biology-14-00526-s001.zip › Table S1.pdf]

Supplementary Table S1. The information on the sequences of the primers used in this study.

Gene Name	Sequence Product
Fgf21-F2	TCATCTGCCTGGTCTTGGTC
Fgf21-R2	GCTTCTCCATCTTCTCGGTGTT
Vezf1-F	GCAGGTAGAGACACTGAGACTGTGG
Vezf1-R	TGAATGGAGTAGTGAGAGTCACTGG
Map3k1-F	GTCATCGTCTAGCACATCCACATC
Map3k1-R	GCAGCCATCTTCACACACAGTC
GAPDH-F	CACGATGGAGGGGCCGGACTCATC
GAPDH-R	AAAGACCTCTATGCCAACACAGT
mmu-miR-423-5p-F	ACGTGAGGGGCAGAGAGC
mmu-miR-423-5p-R	GTGCAGGGTCCGAGGT
mmu-miR-186-5p-F	CTCCAACGCAAAGAATTCTCC
mmu-miR-186-5p-R	TATGCTTGTTCTCGTCTCTGTGTC
mmu-miR-409-5p-F	ACGATTAGGTTACCCGAGCAA
mmu-miR-409-5p-R	TATGGTTCTTCACGACTCCTTCAC
mmu-miR-127-3p-F	ATTACTTCGGATCCGTCTGAGC
mmu-miR-127-3p-R	TATGGTTTTGACGACTGTGTGAT
mmu-miR-134-5p-F	CCTCTATTCTGTGACTGGTTGACC
mmu-miR-134-5p-R	TATGGTTTTGACGACTGTGTGAT
mmu-miR-381-3p-F	GGCATATACAAGGGCAAGCT
mmu-miR-381-3p-R	TATGGTTTTGACGACTGTGTGAT
mmu-miR-152-3p-F	GTAACTCGCTCAGTGCATGACA
mmu-miR-152-3p-R	TATGGTTGTAGACGACTCCTTGAC
mmu-miR-434-5p-F	AGATCACGCTCGACTCATGGT
mmu-miR-434-5p-R	TATGGTTGTTCACGACTGGTTCAC
mmu-miR-369-3p-F	CAATGGAAATCGAATAATACATGG
mmu-miR-369-3p-R	TATGCTTGTTCTCGTCTCTGTGTC
mmu-miR-136-5p-F	CGGCGAACTCCATTTGTTT
mmu-miR-136-5p-R	TATGCTTGTTCTCGTCTCTGTGTC
U6-F	CAGCACATATACTAAAATTGGAACG
U6-R	ACGAATTTGCGTGTCATCC
Target gene Vezf1-F	TCGAGCAAATTAAAGTTAACCAGTCACAT
Target gene Vezf1-R	CTAGATGTGACTGGTTAACTTTAATTTGC
Target gene Map3k1-F	TCGAGGTTCAGATCAGCTCTAATGGAGAT
Target gene Map3k1-R	CTAGATCTCCATTAGAGCTGATCTGAACC
MUT Vezf1-F	TCGAGCAAATTAAAGTTAACTCTATCACT
MUT Vezf1-R	CTAGAGTGATAGAGTTAACTTTAATTTGC
MUT Map3k1-F	TCGAGGTTCAGATCAGCTCTTTACAGAGT
MUT Map3k1-R	CTAGACTCTGTAAAGAGCTGATCTGAACC
